# An Innovative and Efficient Low Temperature Hydrothermal-Calcination Process for β-FeOOH Nanorods and Hollow-Structure α-Fe_2_O_3_ Magnetic Nanorods Exclusively Utilizing FeCl_3_ in an Alcohol-Containing Aqueous Solution

**DOI:** 10.3390/ma18092079

**Published:** 2025-05-01

**Authors:** Lei Sun, Zhou Wang, Ruijiang Liu

**Affiliations:** 1 School of Pharmacy, Jiangsu University, Zhenjiang 212013, China; dyleisun@163.com; 2 College of Vanadium and Titanium, Panzhihua University, Panzhihua 617000, China; pzhwangzhou@163.com

**Keywords:** α-Fe_2_O_3_ nanorods, hollow-structure, hydrothermal-calcination approach, β-FeOOH nanorods

## Abstract

A facile low temperature hydrothermal-calcination approach was developed for the fabrication of β-FeOOH nanorods (NRs) and hollow-structure α-Fe_2_O_3_ magnetic nanorods (MNRs), and the products were characterized using SEM, TEM, XRD and VSM techniques. To achieve smaller-sized β-FeOOH NRs, the effects of Fe^3+^ concentration, the volume ratio of ethanol to water in solution, hydrothermal temperature, and hydrothermal time on the structure of the precursors were systematically investigated, and the nanorods with an average length 104 nm and diameter 36 nm were fabricated at hydrothermal temperature of 100 °C for 2 h using 0.15 M ferric chloride hexahydrate in 50% ethanol solution. Subsequently, the hollow-structure α-Fe_2_O_3_ MNRs with an average length of 67 nm, diameter of 20 nm, and thickness of 5 nm were successfully obtained via the calcination process at 400 °C for 2.5 h for versatile applications.

## 1. Introduction

Nanoscale materials have gained widespread applications in various fields due to their unique properties [1,2]. In particular, the iron oxide nanomaterials have garnered significant attentions due to their facile preparation, abundant availability, the reduced environmental impact, and their remarkable characteristics, such as small crystal size, large specific surface area, modifiable surfaces, exceptional magnetic properties, and excellent biocompatibility [3,4,5], These features endow them with promising potentials for numerous practical applications [6,7,8]. During application processes, iron oxide nanorods reveal significant potentials. For instance, iron oxide nanorods can carry more drugs and more easily penetrate cells [9]; Additionally, iron oxide nanorods can provide increased interspace to enhance the gas sensing performance [10].

To further enhance the application potentials of nanorods, including low density, expansive specific surface area, excellent thermal insulation properties, and a high load-bearing capacity [11,12], the hollow inorganic nanorods have recently attracted significant attention, and demonstrated promising applications in various fields, including water treatment [13], photoelectric sensor [14], drug delivery [15], building materials [16], electrochemical capacitor [17], and others. For example, in the field of electrochemistry, hollow nanostructures are highly appealing as electrocatalysts because they can significantly improve the utilization efficiency of the catalyst, facilitate transport of the electroactive materials, and effectively mitigate issues such as catalyst aggregation, dissolution, and Ostwald ripening [18]; in the sensor domain, the hollow nanostructures can notably enhance gas sensing performance [19].

The formation of hollow structures is the critical factor in the development of hollow nanomaterials. To date, numerous techniques have been developed for fabricating inorganic hollow nanostructures, such as the sol-gel method [20], electrostatic adsorption followed by annealing method [21], among others. Nevertheless, these methods tend to be tedious and complex, frequently requiring the use of multiple hazardous chemicals.

In this work, an innovative hydrothermal-calcination combination approach was developed, and hollow-structure α-Fe_2_O_3_ magnetic nanorods (MNRs) were successfully fabricated and characterized. This method creatively used only ferric chloride and ethanol solution through hydrothermal-calcination process, the utilization of ethanol not only effectually moderated the hydrothermal reaction conditions but also provided an efficient pathway for controlling preparation the size of the nanorods [22]. The fabrication of hollow α-Fe_2_O_3_ MNRs with diverse morphologies was deemed particularly appealing by virtue of adaptability for any possible application scenarios. Therefore, after confirming the feasibility of the fabrication strategy, extensive investigations were carried out to investigated the effects of ferric ion concentration, ethanol solution concentration, hydrothermal temperature, hydrothermal temperature, and calcination conditions, aiming to achieve precise control over the preparation of hollow α-Fe_2_O_3_ MNRs.

## 2. Materials and Methods

### 2.1. Fabrication of β-FeOOH Nanorods

The β-FeOOH nanorods (NRs) were fabricated via a hydrothermal process. Specifically, various amounts of FeCl_3_·6H_2_O (AR, 99.0%) ranging from 0.546 g to 4.368 g were dispersed in 80 mL alcohol (AR, 99.7%) containing aqueous solutions under magnetically stirring to generate different concentrations of Fe^3+^ mixtures (0.025 M, 0.05 M, 0.1 M, 0.15 M, and 0.2 M). The volume ratios of alcohol to water were adjusted to prepare solutions with varying alcohol concentrations (75%, 62.5%, 50%, 37.5%, 25%, and 12.5%). Subsequently, the Fe^3+^ mixtures were severally transferred into 100 mL Teflon coated stainless steel reactors. These sealed reactors were then placed in the programmable furnaces (KSL-1200X-J, Kejing, Hefei, China) and maintained at specific temperatures (80 °C, 100 °C, 120 °C, or 140 °C) for predetermined durations (0.5 h, 1 h, 2 h, or 3 h). After cooling, the resulting turbid liquids were centrifuged at 10,000 rpm, and the precipitates were washed repeatedly with ethanol or water before undergoing ambient drying at 60 °C.

### 2.2. Preparation of Hollow α-Fe_2_O_3_ MNRs

The hollow-structure α-Fe_2_O_3_ MNRs were prepared via the calcination process using β-FeOOH NRs as the precursors. Typically, 0.5 g of β-FeOOH precursor was placed into a 50 mL crucible, and the crucible containing the β-FeOOH NRs was calcined at 400 °C for 2.5 h with the heating rate of 3 °C/min. After the calcination process, and the furnace was naturally cooled to ambient temperature, the resulting solid product was finely ground to obtain the hollow-structured α-Fe_2_O_3_ MNRs.

### 2.3. Characterization of β-FeOOH NRs and Hollow α-Fe_2_O_3_ MNRs

The morphology and composition of β-FeOOH NRs and hollow α-Fe_2_O_3_ MNRs were examined using scanning electron microscope (SEM, JSM-TOOIF, JEOL, Showa, Kyoto, Japan) High-Resolution Transmission Electron Microscopy (HRTEM, JEM-2100, JEOL, Japan), transmission electron microscopy (TEM, JEM-2100, JEOL, Japan), and Fourier Transform Infrared Spectroscopy (FTIR, Nicolet IS 10, Thermo, Waltham, MA, USA), their X-ray diffraction (XRD, D8 Advance, BRUKER, Karlsruhe, Germany) patterns were characterized by Rigaku D/max 2500 PC X-ray diffraction from 20° to 80° with Cu-Kα radiation for phase identification; their magnetic measurements were taken on a vibrating sample magnetometer (VSM, ADE DMS-HF-4, LakeShore, Columbus, OH, USA). Thermogravimetric (TG) analysis of β-FeOOH NRs during calcination was carried out by Synchronous thermal analyzer (STA 449 F5, Netzsch, Selb, Bavaria, Germany).

## 3. Results

### 3.1. Characteristics of β-FeOOH NRs

**Figure 1:**
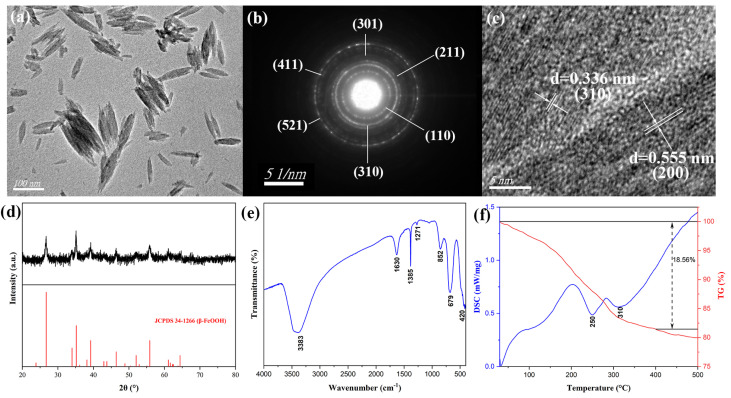
TEM micrograph (**a**), SAED image (**b**), HRTEM image (**c**), XRD pattern (**d**), FTIR spectrum (**e**), and TG-DSC analysis (**f**) of β-FeOOH NRs fabricated at the hydrothermal temperature of 100 °C for 2 h using ferric chloride of 0.15 M in 50% alcohol-containing aqueous solution.

The structure and morphology characteristics of β-FeOOH precursors fabricated at the hydrothermal temperature of 100 °C for 2 h using ferric chloride of 0.15 M in 50% alcohol-containing aqueous solution were displayed as the TEM micrograph (Figure 1a), which indicated that the β-FeOOH precursors were nanorods with an average length of 104 nm and diameter of 36 nm. The corresponding selected area electron diffraction (SAED) image was presented in Figure 1b, and all the diffraction rings were labeled with their values of diffraction crystal surface. The HRTEM image for β-FeOOH NRs was revealed in Figure 1c, the β-FeOOH crystals reflected obvious lattice structure, two lattices obviously appeared, the lattice widths were 0.336 nm and 0.555 nm, which corresponded to the (310) and (200) lattice planes of β-FeOOH crystal, and these data also verified the formation of β-FeOOH NRs. The composition of β-FeOOH NRs was characterized by X-ray diffraction (XRD) as Figure 1d. The diffraction peaks of 2θ corresponding to 26.725°, 34.003°, 35.161°, 39.219°, 46.433, and 55.901° were in accordance with characteristic peaks of Joint Committee Power Diffraction Standard Data card (JCPDS No. 34-1266) of β-FeOOH, which demonstrated the formation of β-FeOOH phase. The well-defined and distinct diffraction peaks unequivocally indicated the presence of a highly pure β-FeOOH phase with excellent crystallinity.

The FTIR spectrum for β-FeOOH NRs was presented in Figure 1e, the diffraction peak at 852 cm^−1^ corresponded to the bending vibration of Fe-OH-Fe, the diffraction peaks at 1271 cm^−1^, 679 cm^−1^ and 420 cm^−1^ were attributed to the bending vibration of Fe-O-H, confirming the formation of β-FeOOH NRs. Additionally, the diffraction peak at 1385 cm^−1^ was a characteristic peak of -CH_3_, indicating the residual presence of alcohol in the product. The diffraction peaks at 3383 cm^−1^ and 1630 cm^−1^ corresponded to the characteristic peak of H_2_O. TG-DSC analysis for β-FeOOH NRs ranging from 30 °C to 500 °C was shown in Figure 1f, DSC curve displayed that there were two larger exothermal stages at around 250 °C and 310 °C, and the most part of weight loss was happened for 18.56%, which revealed β-FeOOH NRs and other substances in the product could be completely decomposed as the heating temperature exceeded 350 °C, therefore, β-FeOOH NRs were calcined at 400 °C for 2.5 h to prepare hollow-structure Fe_2_O_3_ NRs.

### 3.2. Optimization on the Fabrication of β-FeOOH NRs

The morphology of β-FeOOH NRs was influenced by the concentration of Fe^3+^, the purity of ethanol solution, the hydrothermal temperature, and the hydrothermal duration in the hydrothermal process. To achieve β-FeOOH NRs with controllable shape and size, the effects of these conditions were systematically investigated.

#### 3.2.1. Effect of Volume Ratio of Ethanol in Aqueous Solution

The addition of alcohol played a multifaceted role in nanomaterial synthesis, affecting reaction kinetics, morphology control, and functionalization. For example, Quinson et al. [23] found that too low dose of alcohol would lead to non-forming of NPs, while too high would lead to the increased particle size; Panagopoulos et al. [24] reported AuNPs prepared with ethanol instead of other alcohols were smaller; Liu et al. [25] reported that the CNDs synthesized in ethylene glycol fractions were much smaller than those in water. Besides, the increase of alcohol ratio in water-alcohol solution would lead to the sphericity of nanomaterials [26,27]. Also, nanomaterials prepared by ethanol had better biocompatibility than other alcohols [23]. The specific effect of the addition of alcohol on the synthesis of nanomaterials and its mechanism were listed in Table S1.

**Figure 2:**
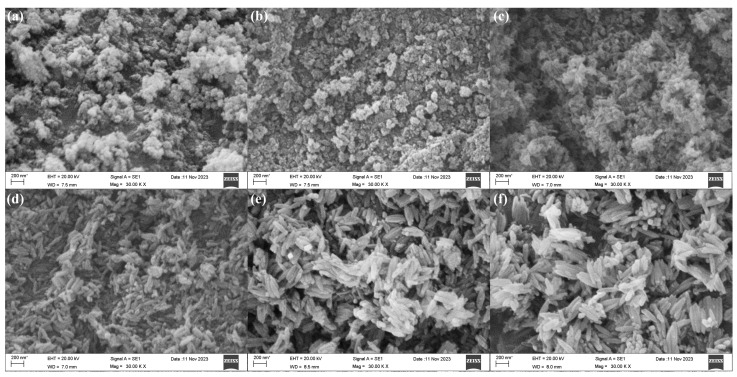
SEM morphologies of β-FeOOH nanomaterials fabricated at the hydrothermal temperature of 120 °C for 2 h using ferric chloride of 0.1 M in various volume ratios of ethanol in aqueous solution: 75% (**a**), 62.5% (**b**), 50% (**c**), 37.5% (**d**), 25% (**e**), 12.5% (**f**).

So, to prepare nanomaterials with better morphology and performance, the effect of volume ratio of ethanol was explored. The SEM morphologies of β-FeOOH nanomaterials fabricated at the hydrothermal temperature of 120 °C for 2 h using ferric chloride of 0.1 M in various volume ratios of ethanol and aqueous solution were presented in Figure 2.

According to SEM observations, the fabrication of β-FeOOH nanomaterials was significantly influenced by the ethanol volume ratio in aqueous solution. The β-FeOOH NRs failed to form when the ethanol volume ratio exceeded or equaled 62.5% as displayed in Figure 2a,b, the larger ethanol volume ratio in aqueous solution significantly impeded the growth rate of the nanorods, diminished the size of the nanomaterials, and resulted in poor orientation. Conversely, with the volume ratio of ethanol in aqueous solution decreasing from 50% to 12.5% (Figure 2c–f), the length of the β-FeOOH NRs significantly increased as 137 nm at 50%, 245 nm at 37.5%, 283 nm at 25%, 402 nm at 12.5. Meanwhile, their respective diameters were 53 nm, 85 nm, 90 nm, and 105 nm, respectively. Obviously, ethanol in aqueous solution well played the effect of the restricted growth. Therefore, with the increase of ethanol in aqueous solution, the size of nanomaterials became smaller, even the nanorods couldn’t be formed.

#### 3.2.2. Effect of Ferric Ion Concentration

In the hydrothermal process, the solution dissolution equilibrium was obligatory. It was crucial to determine the optimal degree of supersaturation for efficient crystal growth. The elevated concentration of reactant could lead to a substantial level of solution supersaturation, which directly impacted both nucleation and growth rate of crystal, thereby influencing the morphology and dimension. The concentration of ferric ion involved in the reaction determined the equilibrium process of the hydrolysis reaction and the nucleation process.

**Figure 3:**
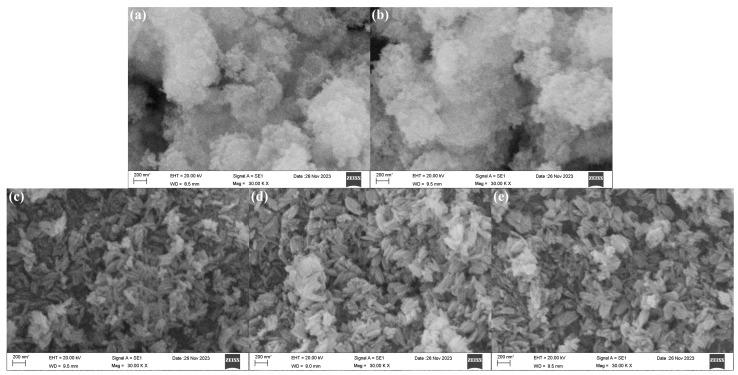
SEM morphologies of β-FeOOH nanomaterials fabricated at the hydrothermal temperature of 120 °C for 2 h using ferric chloride of 0.025 M (**a**), 0.05 M (**b**), 0.1 M (**c**), 0.15 M (**d**), and 0.2 M (**e**) in 50% alcohol-containing aqueous solution.

The SEM morphologies of β-FeOOH NRs fabricated at hydrothermal temperature of 100 °C for 2 h using various concentrations of ferric chloride (0.025–0.1 M) in 50% alcohol-containing aqueous solution were revealed in Figure 3. Obviously, when the concentrations of ferric chloride were 0.025 M and 0.05 M, the β-FeOOH NRs could not be formed, only tiny spherical nanoparticles were formed (Figure 3a,b). When the concentration of ferric chloride reached 0.1 M, the rod-like β-FeOOH began to be formed. The reason might be that the insufficient concentration of ferric ion hindered the formation of precipitate nuclei, resulting in spherical structure. Conversely, an increased concentration of ferric chloride promoted a more comprehensive reaction between iron ions and hydroxide ions, leading to the formation of uniform and larger nanorods. However, the impact of ferric ion concentration on morphologies of β-FeOOH NRs with the three similar rod-like morphologies was reduced once the rod-like structure β-FeOOH NRs were formed (Figure 3c–e). The average lengths of the nanorods fabricated with various concentrations of ferric ion (0.1 M, 0.15 M, and 0.2 M) were 217 nm, 197 nm, and 208 nm, respectively; and the corresponding average diameters were 68 nm, 62 nm, and 70 nm, respectively.

Thus, it could be seen that, during the preparation of β-FeOOH NRs, the concentration of ferric chloride affected the size and morphology of the nanorods. Clearly, the formation of β-FeOOH NRs was hindered in the solution of ferric ion concentration below 0.1 M, and the presence of higher ferric ion concentration was the necessary condition for the formation of β-FeOOH NRs. At the same time, during the concentration of ferric ion changing from 0.1 M to 0.2 M, the average length and average diameter of β-FeOOH NRs reached minimum of 197 nm and 62 nm when the concentration of ferric ion was 0.15 M, therefore, 0.15 M was selected for the concentration of ferric ion to fabricate β-FeOOH NRs.

#### 3.2.3. Effect of Hydrothermal Time

The hydrothermal time was widely recognized as a crucial determinant of the nucleation and growth dynamics of crystals during the hydrothermal process. Inadequate hydrothermal duration might lead to incomplete crystal formation, whereas excessive hydrothermal duration could result in the emergence of secondary crystal phases and the overgrowth of nanomaterials. The degree of crystallinity increased with time, as the hydrothermal process facilitated the gradual growth of crystal cores into oriented grains. During the hydrothermal process, the reaction mixture was subjected to the elevated temperatures and pressures within a hermetically sealed system. Under these stringent conditions, the components could dissolve in the solvent, resulting in the formation of supersaturated solution. As the temperature decreased, the supersaturated solution became unstable and began to precipitate out as solid crystals. Initially, the small crystal nuclei formed within the solution, serving as seeds for further crystal growth. Over time, these seed crystals grew larger through continuous deposition of the dissolved materials onto their surfaces. The solid crystals were gradually formed in the supersaturated solutions as a result of the decrease of hydrothermal temperature, which commenced with the emergence of minute crystal nuclei. These crystal nuclei served as nucleation sites for subsequent crystal growth, gradually enlarging through continuous deposition of dissolved material onto their surfaces.

**Figure 4:**
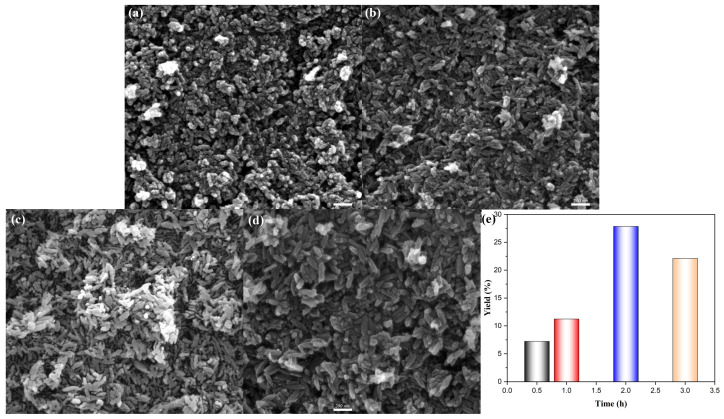
SEM morphologies of β-FeOOH NRs fabricated at hydrothermal temperature of 100 °C for hydrothermal time of 0.5 h (**a**), 1 h (**b**), 2 h (**c**), 3 h (**d**), and the corresponding yields (**e**) of β-FeOOH NRs using ferric chloride of 0.15 M in 50% alcohol-containing aqueous solution.

Figure 4 represented the SEM morphologies of β-FeOOH NRs fabricated at hydrothermal temperature of 100 °C for various durations ranging from 0.5 h to 3 h. Initially, β-FeOOH NRs fabricated at 100 °C for 0.5 h failed to form properly, as shown in Figure 4a, which could be attributed to the insufficient hydrothermal reaction time; In contrast, β-FeOOH NRs fabricated at 100 °C for hydrothermal durations of 1–3 h presented well-defined rod-like morphology. Specifically, when the hydrothermal duration was 1 h, the average length and diameter of β-FeOOH NRs with lower yield (Figure 4e) were measured at 100 nm and 34 nm (Figure 4b). Similarly, the β-FeOOH NRs fabricated with the hydrothermal duration of 2 h exhibited comparable dimension with average length of 104 nm and average diameter of 36 nm (Figure 4c), but the yield was 2.5 times of that for the hydrothermal duration of 1 h (Figure 4e). While, a further increase in hydrothermal duration of 3 h led to the significant growth of β-FeOOH NRs (Figure 4d) with their average length and average diameter of 161 nm and 53 nm, respectively; obviously, the further prolongation of the hydrothermal duration resulted in the speedy magnification of sizes.

The minimal difference in size and significant disparity in yield could be attributed to the fact that not all crystal nuclei within the entire container system underwent simultaneous growth in the low hydrothermal temperature process. The energy expended during the initial two hours tended to predominantly utilized for the formation of new nuclei, rather than being deposited onto pre-existing larger nuclei to augment their size. As time progressed, the lower concentration of the dissolved material tended to deposit preferentially in the existing crystal core rather than forming new ones. However, this trend became more pronounced with prolonged reaction time.

#### 3.2.4. Effect of Hydrothermal Temperature

**Figure 5:**
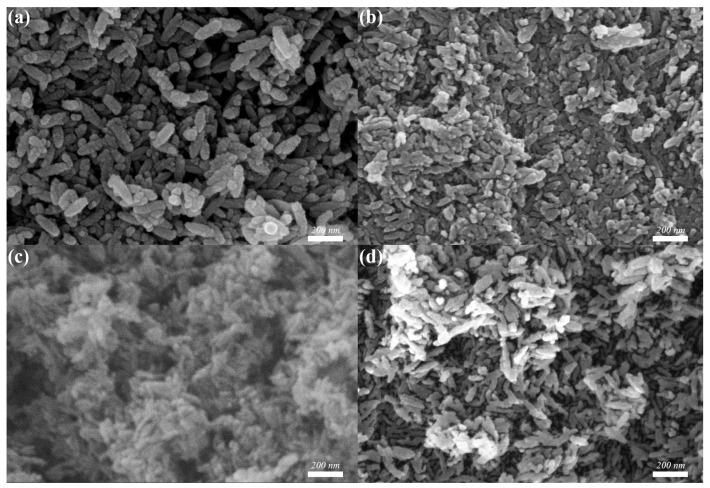
SEM morphologies of β-FeOOH NRs fabricated at hydrothermal temperature of 80 °C (**a**), 100 °C (**b**), 120 °C (**c**), and 140 °C (**d**) for 2 h using ferric chloride of 0.15 M in 50% alcohol-containing aqueous solution.

The hydrothermal temperature played a crucial role in the hydrothermal process for the fabrication of nanomaterials [28,29,30]. And as it was widely acknowledged that ethanol possessed a significantly lower boiling point compared with water, the introduction of ethanol as an additive in hydrothermal reaction led to a pressure increase in the reaction vessel, thereby facilitating and promoting the reaction. The SEM images of β-FeOOH NRs fabricated at different hydrothermal temperatures for 2 h using ferric chloride of 0.15 M in 50% alcohol-containing aqueous solution were shown in Figure 5. Obviously, the β-FeOOH NRs were successfully fabricated even at the hydrothermal temperature as low as 80 °C, the as-fabricated β-FeOOH NRs exhibited an average length of 163 nm and an average diameter of 37 nm (Figure 5a).

The average lengths of β-FeOOH NRs fabricated at 100 °C, 120 °C, and 140 °C were 104 nm, 106 nm, and 111 nm, respectively; while, the corresponding average diameters were determined to be 36 nm, 37 nm, and 41 nm, as shown in Figure 5b–d. Obviously, the size of β-FeOOH NRs significantly decreased with the hydrothermal temperature decreasing form higher temperature to the boiling point of water, and the effect of hydrothermal temperature subsequently exhibited a diminished tendency with the decrease of it. Considering the pursuit of smaller sizes for better nanomaterial applications, the hydrothermal temperature of 100 °C was employed for the fabrication of β-FeOOH NRs.

### 3.3. Preparation and Formation Mechanism of Hollow α-Fe_2_O_3_ NRs

**Figure 6:**
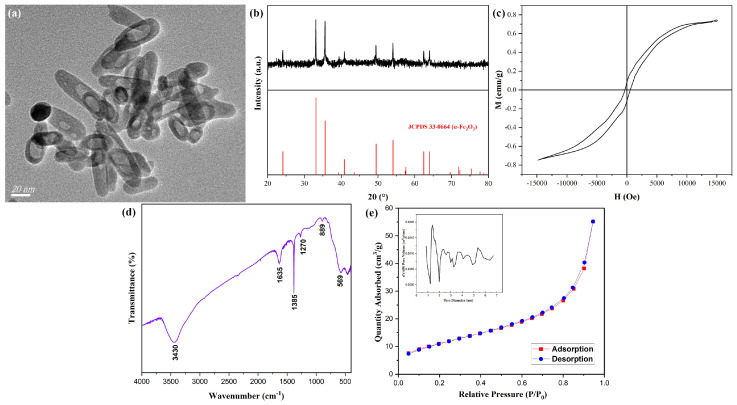
TEM image (**a**), XRD pattern (**b**), hysteresis loops (**c**), FTIR spectrum (**d**), and Nitrogen adsorption and desorption curves (**e**) of hollow α-Fe_2_O_3_ NRs.

Calcination was a widely employed approach for the preparation of nanomaterials with hollow, mesoporous, or tubular architectures [31,32,33]. β-FeOOH NRs fabricated at 80 °C for 2 h using ferric chloride of 0.15 M in 50% alcohol-containing aqueous solution were calcined at 400 °C for 2.5 h with the heating rate of 3 °C/min to obtain hollow α-Fe_2_O_3_ MNRs, the TEM image of hollow α-Fe_2_O_3_ MNRs was shown in Figure 6a, the formation of the distinct hollow structure was evident in comparison with Figure 1a, their average length, diameter, and thickness were about 67 nm, 20 nm, and 5 nm, respectively; their structure and the size were similar to those of literature [22]. The XRD pattern of hollow α-Fe_2_O_3_ MNRs (shown in Figure 6b) revealed that the diffraction angles and proportions of hollow α-Fe_2_O_3_ MNRs were consistent with Joint Committee Power Diffraction Standard Data Card (JCPDS No. 33-0664) of α-Fe_2_O_3_, thereby confirming the presence of α-Fe_2_O_3_ phase. Figure 6c showed the hysteresis loops of hollow α-Fe_2_O_3_ MNPs, the saturation magnetization (Ms) reached 0.73 emu/g. Additionally, the relatively low residual magnetism and magnetic coercive force indicated their soft magnetic characteristic.

The FTIR spectrum for α-Fe_2_O_3_ MNRs was shown in (Figure 6d), the characteristic peak of Fe-O appeared at 569 cm^−1^, revealing the formation of α-Fe_2_O_3_ MNRs, the other peaks were basically owing to the water in sample. The Nitrogen adsorption and desorption curves onto α-Fe_2_O_3_ MNRs were displayed in Figure 6e, the adsorption type belonged to III type, revealing the macropore character. The pore volume analysis image was set into a corner of Figure 6e, the distribution size of pore mainly was 1–6 nm, and the calculated specific surface area was 41.2 m^2^/g, the lager value for the inorganic nanomaterials suggested the hollow structure.

The formation mechanism of hollow α-Fe_2_O_3_ NRs could be divided into stages: (1) The template formation of α-Fe_2_O_3_ NRs. When the β-FeOOH NRs were placed into programmed temperature furnace, the calcination began with larger heating rate, in the short time, the β-FeOOH nanomaterials on the surfaces of β-FeOOH NRs had translated into the hard α-Fe_2_O_3_ component, the template of α-Fe_2_O_3_ NRs was formed. (2) The formation of hollow structure. With the continue heating, the heat was transmitted into the interior of β-FeOOH NRs, the inner β-FeOOH NRs also began to translate into α-Fe_2_O_3_ component, however, the generated α-Fe_2_O_3_ only could adhere onto outer template, resulting in the increase of the thickness for α-Fe_2_O_3_ template, at the same time, with the decomposition of β-FeOOH component, -OH disappeared, the disappearance of component inevitably led to formation of hollow structure.

## 4. Conclusions

In this project, hollow α-Fe_2_O_3_ MNRs were successfully prepared via low temperature hydrothermal-calcination process.

(1) β-FeOOH NRs were fabricated via the hydrothermal process, β-FeOOH NRs fabricated at the hydrothermal temperature of 100 °C for 2 h using ferric chloride of 0.15 M in 50% alcohol-containing aqueous solution reached minimum sizes with an average length of 104 nm and an average diameter of 36 nm.

(2) The hollowα-Fe_2_O_3_ MNRs were further successfully fabricated with β-FeOOH NRs as the precursors through the calcination process. The hollow-structure α-Fe_2_O_3_ MNRs calcined at 400 °C for 2.5 h with the heating rate of 3 °C/min revealed the obvious hollow structure, and their average length, diameter, and thickness were about 67 nm, 20 nm, and 5 nm, respectively; and their saturation magnetization and specific surface area reached 0.73 emu/g and 41.2 m^2^/g, respectively.

## Data Availability

The original contributions presented in this study are included in the article. Further inquiries can be directed to the corresponding author.

## Supplementary Materials

The following supporting information can be downloaded at: https://www.mdpi.com/article/10.3390/ma18092079/s1, Table S1: The effect of the addition of alcohol on the synthesis of nanomaterials.
