# Supplementary material for: An Innovative and Efficient Low Temperature Hydrothermal-Calcination Process for β-FeOOH Nanorods and Hollow-Structure α-Fe2O3 Magnetic Nanorods Exclusively Utilizing FeCl3 in an Alcohol-Containing Aqueous Solution
Source: Materials (Basel). 2025 May 1;18(9):2079. doi: 10.3390/ma18092079 (PMC12073027; doi:10.3390/ma18092079)
Supplement: Supplementary file 1 [file materials-18-02079-s001.zip › materials-3606204-supplementary.pdf]

Supplementary Material

An Innovative and Efficient Low Temperature Hydrothermal-Calcination Process for β-FeOOH Nanorods and Hollow-Structure α-Fe_2O_3 Magnetic Nanorods Exclusively Utilizing $FeCl_3$ in an Alcohol-Containing Aqueous Solution

Lei Sun [1], Zhou Wang [2] and Ruijiang Liu [1,*]

[1] School of Pharmacy, Jiangsu University, Zhenjiang 212013, China; dyleisun@163.com
[2] College of Vanadium and Titanium, Panzhihua University, Panzhihua 617000, China; pzhwangzhou@163.com
* Correspondence: luckystar_lrj@ujs.edu.cn

Table S1. The effect of the addition of alcohol on the synthesis of nanomaterials.

Modified Property	Modification Direction	Mechanism	Ref.
Particle Size	At ratio of alcohol and water <30 vol %, no NPs formed; At higher values, larger NPs were obtained or no stable colloids.	The reaction required balancing adequate reducing agents to enable progress while preventing nanoparticle overgrowth from excess reductants.	[23]
	Using ethanol instead of methanol led to smaller NPs, and mono-alcohols with higher molecular weights and larger alkyl chains didn't lead to small NPs.	Compared with methanol or mono-alcohols, nucleation events of Au clusters in ethanol were fast relative to the growth of the NPs from these clusters.	[24]
	The CNDs synthesized in 50 and 100% ethylene glycol fractions were much smaller than those in water.	Alcohols could cap the CNDs and inhibit their agglomeration resulting in a better dispersion.	[25]
Morphology	ZnO NPs tended to form submicrometer spherical depend on the used type of alcohol.	ZnO NPs minimized the surface energy by forming spherical aggregates with the lowest surface-to-volume ratio possible.	[26]
	Changing the reaction medium from water, alcohol-water to alcohol, the morphology changed from big irregular nanosheets, microspheres, leaf-like nanosheets, and nanoparitcles to big microspheres.	The increase of the alcohols dose led to large numbers of homogeneous nucleation at once and resulted in the formation of microspheres by Ostwald ripening in the latter hydrothermal procedure.	[27]
Biocompatibility	Using ethanol instead of methanol led to lower toxicity and higher environmental friendliness.	Ethanol demonstrated low toxicity and rapid biodegradability, minimizing residual hazardous byproducts and ecological contamination risks in synthetic applications.	[23]

References

23. Quinson, J.; Aalling-Frederiksen, O.; Dacayan, W.L.; Bjerregaard, J.D.; Jensen, K.D.; Jørgensen, M.R.V.; Kantor, I.; Sørensen, D.R.; Kuhn, L.T.; Johnson, M.S.; Escudero-Escribano, M.; Simonsen, S.B.; Jensen, K.M.O. Surfactant-Free Colloidal Syntheses of Gold-Based Nanomaterials in Alkaline Water and Mono-Alcohol Mixtures. *Chem. Mater*. **2023**, 35, 2173-2190.

24. Panagopoulos, D.; Alamdari, A.A.; Quinson, J. Surfactant-Free Colloidal Gold Nanoparticles: Room Temperature Synthesis, Size Control and Opportunities for Catalysis. *Mater. Today Nano* **2025**, 29, 100600.

25. Liu, D.; Qu, F.; Zhao, X.; You, J. Generalized One-Pot Strategy Enabling Different Surface Functionalizations of Carbon Nanodots to Produce Dual Emissions in Alcohol–Water Binary Systems. *J. Phys. Chem. C* **2015**, 119, 17979–17987.

26. Šarić, A.; Despotović, I.; Štefanić, G. Alcoholic Solvent Influence on ZnO Synthesis: A Joint Experimental and Theoretical Study. *J. Phys. Chem. C* **2019**, 123, 29394–29407.

27. Chen, M.; Gao, L. Synthesis of Leaf-like Ag_2S Nanosheets by Hydrothermal Method in Water–Alcohol Homogenous Medium. *Mater. Lett*. **2006**, 60, 1059–1062.
